# Postpartum hemorrhage: Findings of a global survey by the World Association of Trainees in Obstetrics and Gynecology (WATOG)

**DOI:** 10.1002/ijgo.70512

**Published:** 2025-09-11

**Authors:** Akaninyene E. Ubom, Zahra Muslim, Jolly Beyeza‐Kashesya, Dietmar Schlembach, Zechariah J. Malel, Ferdousi Begum, Inês Nunes, Alison Wright, FIGO Childbirth and PPH Committee

**Affiliations:** ^1^ PAMO University of Medical Sciences Port Harcourt Nigeria; ^2^ World Association of Trainees in Obstetrics and Gynecology (WATOG) Paris France; ^3^ Riphah International University Faisalabad Pakistan; ^4^ Mulago Specialised Women and Neonatal Hospital Kampala Uganda; ^5^ Vivantes Network of Health GmbH, Clinicum Berlin‐Neukoelln Clinic for Obstetric Medicine Berlin Germany; ^6^ Department of Obstetrics and Gynecology, School of Medicine University of Juba Juba Republic of South Sudan; ^7^ Association of Gynecologists and Obstetricians of South Sudan (AGOSS) Juba Republic of South Sudan; ^8^ Department of Obstetrics & Gynecology Institute of Woman and Child Health Dhaka Bangladesh; ^9^ Department of Obstetrics and Gynecology Gaia/Espinho Local Health Unit Porto Portugal; ^10^ CINTESIS—Center for Health Technology and Services Research University of Porto Porto Portugal; ^11^ Department of Medical Sciences University of Aveiro Aveiro Portugal; ^12^ Royal Free London NHS Foundation Trust London UK; ^13^ University College London London UK

**Keywords:** maternal mortality and morbidity, obstetric hemorrhage, postpartum hemorrhage

## Abstract

Postpartum hemorrhage (PPH) remains the leading cause of maternal mortality globally. This global survey was conducted to identify any disparities in the causes, prevalence, treatment, and mortality burden of PPH, with the aim of proposing relevant recommendations to bridge these disparities and ultimately reduce the global maternal mortality and morbidity burden of PPH. A cross‐sectional survey of maternity care providers worldwide was conducted by the World Association of Trainees in Obstetrics and Gynecology (WATOG) in collaboration with International Federation of Gynecology and Obstetrics (FIGO) Childbirth and PPH Committee. The study instrument was a 15‐item structured electronic questionnaire, designed using Google Forms®. The questionnaire included multiple choice and short answer questions on the baseline characteristics of respondents, causes, prevalence, treatment modalities, and mortality from PPH. The questionnaire was electronically distributed via WATOG and FIGO social media channels to study participants. In total, 339 responses were received from 64 countries in six regions, including Africa, Asia, Europe, North America, South America, and Oceania. The majority (*n* = 182, 53.7%) of respondents reported seeing an average of at least 10 cases of PPH in their hospitals each month. More respondents in low‐ and middle‐income countries (LMICs) in Africa and Asia reported seeing more than 10 PPH cases monthly, compared to those in high‐income countries in Europe and America (57.1% vs. 49.2%, *P* < 0.001). Most (*n* = 318, 93.8%) respondents volunteered that their hospitals recorded less than five PPH‐related maternal mortalities monthly. All (*n* = 8, 2.4%) respondents who reported more than five PPH‐related maternal mortalities were based in LMICs in Africa. Only 133 (39.2%) respondents reported availability of the non‐pneumatic anti‐shock garment (NASG) in their hospitals. Of those who reported non‐availability of the NASG, 60% were in LMICs. The most common treatment for intractable PPH were uterine compression sutures (*n* = 177, 52.2%) and hysterectomy (*n* = 128, 37.8%). Less than 1 in 10 (*n* = 30, 8.8%) reported availability of vascular ligation and embolization procedures. PPH remains a significant obstetric complication globally, with a higher morbidity and mortality burden in LMICs. There is an urgent need for concerted global efforts to reduce maternal morbidity and mortality from PPH, especially in LMICs.

## 1 INTRODUCTION

Postpartum hemorrhage (PPH) is the leading cause of maternal mortality globally, accounting for at least 20% of all maternal deaths.^1^ It complicates 1%–10% of births, with an annual incidence of 14 million and 140 000 related mortality,^2^, ^3^ which translates to one maternal death from PPH every 4 min. Significant associated morbidities include severe postpartum anemia requiring blood transfusion, which complicates 1.6 million (12%) cases.^2^ Others are multisystem organ failure, clotting dysfunction, and hysterectomy (in intractable cases) with its subsequent consequence on fertility.^4^ The burden of maternal mortality and morbidity from PPH is disproportionately higher in low‐ and middle‐income countries (LMICs), where up to 25%–43% of maternal deaths are caused by PPH.^5^, ^6^, ^7^ A WHO analysis of 35 197 maternal deaths documented that whereas PPH is responsible for 13.4% of maternal mortality in high‐income countries (HICs), in Asia and Africa, 30.8% and 33.9%, respectively, of maternal deaths are attributed to PPH.^8^ More than 80% of all maternal deaths from PPH occur in sub‐Saharan Africa and south Asia.^9^, ^10^ Furthermore, the risk of maternal death from PPH is 1 in 1000 births in LMICs compared to 1 in 100 000 births in HICs.^4^, ^11^ These highlight significant regional disparities in the morbidity and mortality burden of PPH. To further evaluate these geographical differences, the World Association of Trainees in Obstetrics and Gynecology (WATOG), in collaboration with the International Federation of Gynecology and Obstetrics (FIGO) Childbirth and PPH Committee, conducted this global survey of maternity care providers to explore the causes, prevalence, mortality burden, and treatment interventions for PPH.

We sought to identify country and regional disparities, with the aim of proposing relevant steps and recommendations to bridge these disparities, and ultimately to reduce maternal mortality and morbidity from PPH globally. Despite a significant 40% decline in global maternal mortality ratio (MMR) between 2000 and 2023 from 328 deaths to 197 deaths per 100 000 live births,^12^ achieving the Sustainable Development Goal 3 (SDG 3) target MMR of less than 70 deaths per 100 000 live births by 2030 requires further evaluation of the causes and epidemiology of PPH and maternal mortality, hence this study.

## 2 METHODS

### 2.1 Study design, study duration, and study population

This was a cross‐sectional study conducted in June 2025 by WATOG in collaboration with the FIGO Childbirth and Postpartum Hemorrhage Committee. WATOG is an international association of all obstetrics and gynecology (ObGyn) trainees/residents and early career obstetricians and gynecologists, who are within 10 years of commencement of ObGyn residency training. The study participants were maternity care providers, including ObGyn consultants/attendings, resident doctors, house officers/senior house officers, nurses, and midwives, worldwide.

### 2.2 Sample size determination

The sample size for this study was estimated using the sample size formula for descriptive studies: *n* = *z* ^2^ × *pq*/e^2^,^13^ where *n* = minimum sample size, *z* = standard normal variate, which is 1.96 for a confidence level of 95%, *p* = global prevalence of PPH based on studies that used objective method of blood loss measurement, estimated to be 14.2%,^14^ *q* = 1 − *p*, e = margin of error, which is 0.05 at the 95% confidence level. Substituting these values into the sample size formula gave an approximate sample size of 187. Allowing for an attrition rate of 10%, the calculated minimum sample size was 206, rounded off to 210.

### 2.3 Study instrument and data collection

Google Forms® was used to design a 15‐item structured electronic questionnaire for this study. The questionnaire included multiple choice and short answer questions, and collected data on the baseline characteristics of the respondents, causes, prevalence, treatment modalities, and mortality from PPH. The questionnaire was electronically distributed daily, via WATOG and FIGO social media channels to study participants. Before wide dissemination, the questionnaire was pre‐tested on 10 maternity care providers to ascertain reliability and validity. The questionnaire was set to allow only one response per respondent to optimize the study integrity. Responses were completely anonymized, with no respondent identifier.

### 2.4 Statistical analysis

Data obtained were analyzed using the SPSS Statistics for Windows, version 25 (IBM Corp., Armonk, NY, USA), and results were presented in tables as percentages. *P* < 0.05 was considered statistically significant.

### 2.5 Ethical considerations

All study participants consented to participating in the study. Completing and submitting the survey responses implied consent to participate in the study. Participation in the study was voluntary and no incentive was provided for study participation. Since the study was completely anonymized, with no patient involvement nor interventions, ethical approval was not sought.

## 3 RESULTS

**FIGURE 1:**
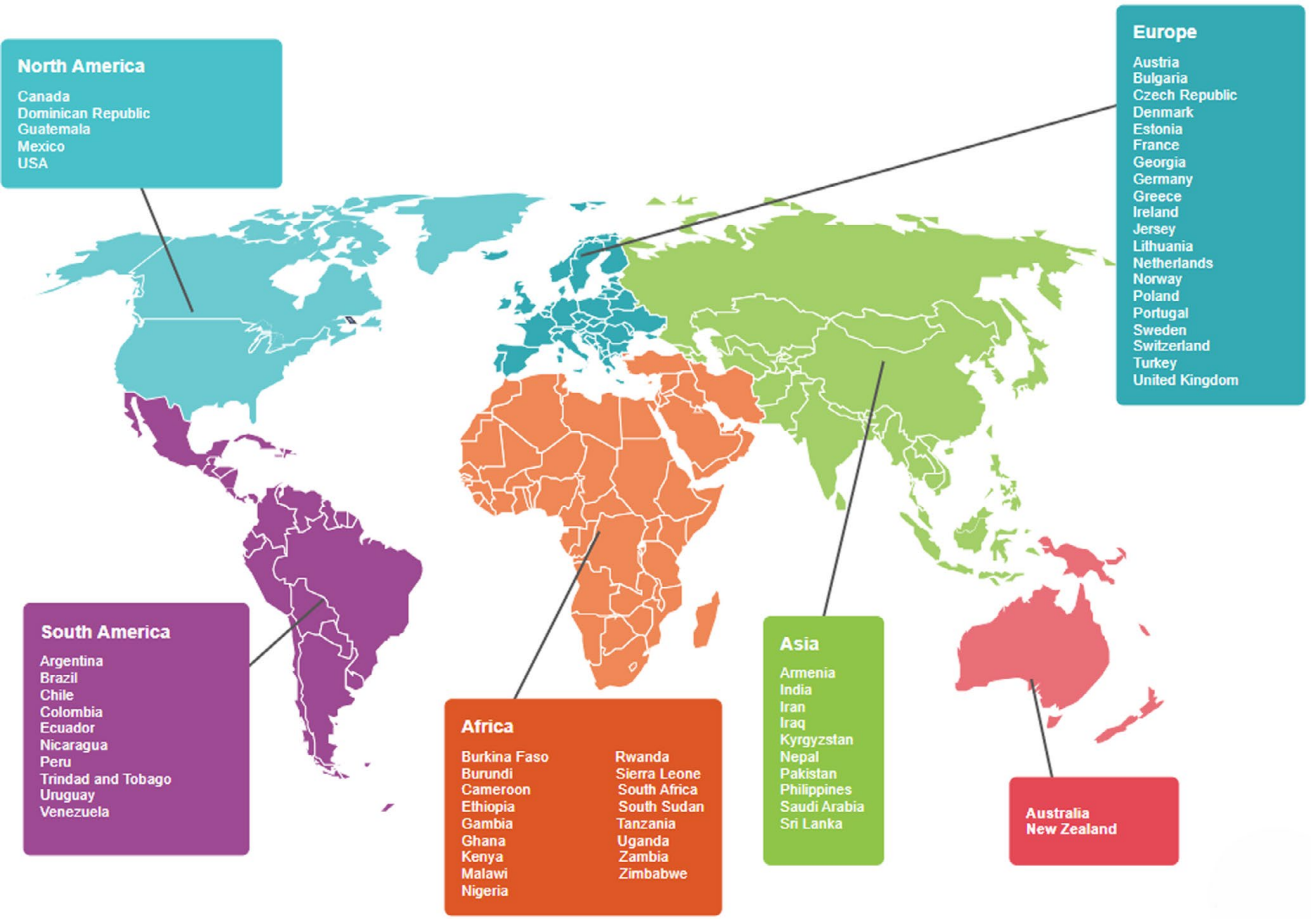
World map showing countries of study respondents.

There were 339 study participants across 64 countries in six regions globally, representing more than 60% of WATOG member countries. The regions included Europe (20 countries), Africa (17 countries), Asia (10 countries), South America (10 countries), North America (5 countries), and Oceania (2 countries) (Figure 1).

### 3.1 Baseline characteristics of the study respondents

The majority of the study respondents were women (*n* = 225, 66.4%), senior resident doctors/senior registrars/specialist registrars (*n* = 141, 41.6%), working in government tertiary hospitals (*n* = 198, 58.4%), residing in middle‐income countries (*n* = 216, 63.7%), and in Africa (*n* = 154, 45.4%) (Table 1).

**TABLE 1:** Baseline characteristics of the study respondents.

Characteristics	*n* = 339^a^
Sex	
Female	225 (66.4)
Male	114 (33.6)
Cadre	
Consultant/Attending	110 (32.5)
Senior Resident Doctor/Senior Registrar/Specialist Registrar	141 (41.6)
Junior Resident Doctor/Registrar	58 (17.1)
House Officer/Senior House Officer	13 (3.8)
Midwife/Nurse	17 (5.0)
Type of hospital	
Tertiary care hospital (government)	198 (58.4)
Secondary care hospital (government)	81 (23.8)
Primary care hospital (government)	6 (1.8)
Private hospital	49 (14.5)
Missionary hospital	5 (1.5)
Country level of income	
High income	95 (28.0)
Middle income	216 (63.7)
Low income	25 (7.4)
Country not indicated by respondent	3 (0.9)
Region	
Africa	154 (45.4)
Europe	69 (20.4)
Asia	40 (11.8)
North/Central America	29 (8.6)
South America	28 (8.2)
Oceania	19 (5.6)

^a^ Values are given as number (percentage).

### 3.2 Causes and prevalence of PPH by country income level and region

Uterine atony was the most common cause of PPH reported in this study (*n* = 313, 92.3%) (Table 2). Genital tract lacerations and retained products of conception were predominantly reported as the most common causes of PPH by respondents in LMICs in Africa and Asia (*n* = 22/26, 84.6%).

**TABLE 2:** Causes, prevalence, and mortality from PPH.

Characteristics	*n* = 339^a^
Average number of births per month in your hospital	
<500	193 (56.9)
500–1000	69 (20.4)
>1000	72 (21.2)
I don't know	5 (1.5)
Average number of PPH cases seen in your hospital monthly	
<10	150 (44.2)
10–20	122 (36.0)
>20	60 (17.7)
I don't know	7 (2.1)
Most common cause of PPH in your hospital	
Uterine atony	313 (92.3)
Genital tract laceration	19 (5.6)
Retained product of conception	7 (2.1)
Average number of maternal mortalities from PPH in your hospital per month	
0	163 (48.1)
1–4	155 (45.7)
5–10	8 (2.4)
I don't know	13 (3.8)

Abbreviation: PPH, postpartum hemorrhage.

^a^ Values are given as number (percentage).

More than one half of the respondents (*n* = 193, 56.9%) reported an average hospital birth rate of less than 500 per month, with at least 10 PPH cases seen monthly (*n* = 182, 53.7%) (Table 2). Over two thirds of respondents who reported 10 or more cases of PPH per month in their hospitals were from LMICs (*n* = 124/182, 68.1%), with more than half in Africa and Asia (*n* = 104/182, 57.1%). On the other hand, less than half of respondents working in hospitals in HICs in Europe and America reported seeing up to 10 PPH cases monthly (*n* = 62/126, 49.2%). The difference was statistically significant (*P* < 0.001).

### 3.3 Maternal mortality from PPH by country income level and region

Respondents predominantly reported that their hospitals recorded less than five maternal mortalities from PPH monthly (*n* = 318, 93.8%), out of which slightly more than half (*n* = 163/318, 51.3%) volunteered zero mortality (Table 2). Only 8 (2.4%) study participants reported more than five maternal mortalities from PPH per month (Table 2), and these were all in hospitals in LMICs in Africa. Of respondents in HICs (*n* = 66/95, 69.5%) and in Europe (*n* = 52/69, 75.4%), 70% and 75%, respectively, reported zero monthly PPH‐related maternal mortality in their hospitals, compared to 40% and less than 35%, respectively, of respondents in LMICs (*n* = 95/241, 39.4%) and in Africa and Asia (*n* = 63/194, 32.5%) (*P* < 0.001).

### 3.4 Treatment modalities for PPH by country income level and region

Only approximately 40% of the study respondents reported availability of a non‐pneumatic anti‐shock garment (NASG) in their hospitals (*n* = 133, 39.2%) (Table 3). The majority of respondents who reported non‐availability of NASGs in their hospitals were from LMICs (*n* = 124/206, 60.2%), with 95 (46.1%) of them in Africa and Asia.

**TABLE 3:** Treatment interventions for PPH.

Characteristics	*n* = 339^a^
Does your hospital have an anti‐shock garment?	
Yes	133 (39.2)
No	206 (60.8)
Most common treatment modality for PPH in your hospital	
Pharmacological	315 (92.9)
Surgical	24 (7.1)
Available pharmacological agents for PPH treatment in your hospital^b^	
Oxytocin	337 (99.4)
Misoprostol	321 (94.7)
Tranexamic acid	319 (94.1)
Ergometrine/methylergometrine/methylergonovine/methergine	204 (60.2)
Carbetocin	169 (49.9)
15 methylprostaglandin F2α/carboprost	85 (25.1)
Prostaglandin E2/sulprostone	3 (0.9)
Syntometrine	2 (0.6)
Intermediary measures routinely used for PPH management in your hospital^b^	
Bimanual compression of the uterus	279 (82.3)
Intrauterine balloon/vacuum/Foley catheter/condom tamponade	275 (81.1)
Uterine packing with gauze	94 (27.7)
Aortic compression	68 (20.1)
Vaginal clamping of uterine arteries	1 (0.3)
Uterine tourniquet	1 (0.3)
Most common surgical procedure for intractable PPH in your hospital^b^	
Uterine compression sutures	177 (52.2)
Hysterectomy	128 (37.8)
Uterine artery ligation	26 (7.7)
Uterine artery embolization	2 (0.6)
Internal iliac artery ligation	1 (0.3)
Ovarian artery ligation	1 (0.3)

Abbreviation: PPH, postpartum hemorrhage.

^a^ Values are given as number (percentage).

^b^ Multiple answers hence *n* > 339.

The predominant treatment modality for PPH was pharmacological (*n* = 315, 92.9%) (Table 3). Less than one third of the respondents reported the availability of facilities for uterine artery embolization (UAE) in their hospitals (*n* = 107, 31.6%). The majority of respondents who reported availability of UAE in their hospitals were from Europe, America, and Oceania (*n* = 77/107, 72.0%) and residing in HICs in these regions (*n* = 62/107, 57.9%). Only one respondent in a missionary hospital in Burundi, Africa, reported availability of UAE.

The most commonly available pharmacological agents for PPH treatment were oxytocin (*n* = 337, 99.4%), misoprostol (*n* = 321, 94.7%), and tranexamic acid (*n* = 319, 94.1%) (Table 3). Only half of the respondents reported availability of carbetocin in their facilities (*n* = 169, 49.9%) and one quarter, 15 methylprostagaldin F2α (carboprost) (*n* = 85, 25.1%) (Table 3). Respondents described that for cases of PPH not responsive to pharmacological treatment, the most commonly used intermediary modalities were bimanual compression of the uterus (*n* = 279, 82.3%) and intrauterine balloon/vacuum/Foley catheter/condom tamponade (*n* = 275, 81.1%) (Table 3).

The most common surgical treatment modalities for intractable PPH included uterine compression sutures (*n* = 177, 52.2%) and subtotal/total hysterectomy (*n* = 128, 37.8%) (Table 3). Less than a tenth (*n* = 30, 8.8%) of respondents reported vascular ligation (including uterine artery ligation, internal iliac artery ligation, and ovarian artery ligation) and UAE as the most common surgical approaches for the treatment of intractable PPH in their hospitals (Table 3). The 3 (0.9%) respondents who reported internal iliac artery ligation and UAE were in HICs in Europe. Most (*n* = 76/95, 80.0%) respondents in HICs reported conservative surgery (including uterine compression sutures, uterine devascularization, and UAE) as the most common treatment for intractable PPH in their hospitals. On the other hand, most respondents who reported hysterectomy as the most common treatment for intractable PPH in their hospitals were in LMICs (*n* = 111/128, 86.7%) and in Africa and Asia (*n* = 104/128, 81.3%).

## 4 DISCUSSION

The majority of respondents in this study reported that their hospitals recorded at least 10 cases of PPH monthly out of an average monthly birth rate of less than 500. This underscores the fact that PPH remains a significant and frequent obstetric complication globally. The frequency of PPH, and PPH‐related mortality, was higher in hospitals in LMICs in Africa and Asia compared to hospitals in HICs in Europe and America. Prevalence, morbidity, and mortality from PPH remains disproportionately higher in LMICs (especially in Africa and Asia) than in HICs.^4^, ^5^, ^6^, ^7^, ^8^, ^9^, ^10^, ^11^ The differences are due to inequalities in healthcare systems, access, and quality of care, worsened by high levels of poverty, poor education, lack of resources, infrastructure, and staffing problems in LMICs.^15^, ^16^ Variations in the methods used to measure blood loss are also contributory: visual estimation versus objective measurement.^3^, ^4^ There are also different definitions and diagnostic criteria for PPH. For instance, while WHO defines PPH as postpartum blood loss in excess of 500 mL,^17^ the American College of Obstetricians and Gynecologists (ACOG) recommends 1000 mL as the diagnostic cutoff.^18^

Oxytocin and misoprostol were the most commonly available uterotonics for the prevention and treatment of PPH in this study. These two drugs may not be optimally effective in many LMICs owing to poor storage conditions.^19^ Oxytocin should be stored at temperatures of 2°C–8°C, as it is degraded at higher temperatures, while misoprostol should be stored in double aluminum blisters, away from humidity.^20^, ^21^, ^22^ These storage conditions are difficult to achieve in many LMICs due to humid tropical climates and erratic power supply. This is in addition to the problem of product adulteration in many LMICs owing to widespread poverty. One study showed that 45.6% of oxytocin supplies in LMICs were of poor quality.^20^ Another study conducted in Nigeria, an LMIC in Africa, revealed that 74.2% and 33.7% of parenteral oxytocin and misoprostol tablets, respectively, were substandard.^21^ This possibly explains the higher frequency of PPH and PPH‐related maternal deaths in LMICs in our study despite widespread availability of oxytocin and misoprostol. For the other uterotonics, only one fourth of our study participants reported availability of carboprost in their hospitals, and only one half, carbetocin, which is heat stable unlike oxytocin. These drugs are not readily available, especially in LMICs, due to their high cost.^19^, ^22^

The widespread availability of tranexamic acid, as reported in this study, is a significantly positive finding. Tranexamic acid is an antifibrinolytic agent that is useful for all cases of PPH irrespective of etiology. Its administration can potentially reduce the need for blood transfusion, incidence of PPH, need for supplementary uterotonics, maternal mortality, and morbidity with minimal side effects.^23^

The NASG is an effective temporizing measure to maintain hemodynamic stability in women with PPH, to allow transfer to a high level of care or until definitive PPH treatment is instituted. FIGO recommends that all healthcare facilities should have a NASG,^24^ as available evidence suggests that it reduces mortality and severe morbidities from PPH.^25^, ^26^ This is, however, not the case. Only 40% of respondents in our study reported availability of the NASG in their hospitals, with 60% of those whose hospitals did not have the NASG being from LMICs. A recent systematic review and meta‐analysis demonstrated that the utilization rate of the NASG in sub‐Saharan Africa was low, at 37%.^27^ Aside from training and knowledge deficits among healthcare providers on the use and application of the NASG, and lack of government support for its implementation, cost is a significant barrier to the widespread adoption of the NASG in LMICs.^28^ The unit price of the NASG is US$ 63,^29^ a significant expense in many low‐resource settings.

Uterine compression sutures were the most commonly reported surgical treatment for intractable PPH in this study. The widespread availability of the skills for uterine compression sutures is especially beneficial in low‐resource settings with a high incidence of PPH among reproductive‐aged women still desiring fertility. Aside from compression sutures, UAE and vascular ligation are conservative surgical techniques recommended for the treatment of PPH when medical and non‐surgical approaches fail.^24^ Less than 10% of our study respondents reported frequent use of these techniques in their hospitals, with only 1% mentioning internal iliac artery ligation and UAE. Internal iliac artery ligation is infrequently performed because it is time‐consuming and technically challenging, with a risk of injury to contiguous structures and variable success rates in the range of 40%–100%.^30^, ^31^, ^32^ UAE requires a trained radiologist and a fully equipped radiology department,^24^ which are lacking in many low‐resource settings, hence its underutilization in these settings. The three respondents that reported internal iliac artery ligation and UAE as common treatment modalities for intractable PPH in this study were in HICs in Europe. With non‐availability of conservative surgical alternatives in many LMICs, hysterectomy becomes the primary surgical approach for treating PPH when medical and non‐surgical options fail, as corroborated in this study. The prevalence of hysterectomy is higher in low‐income countries than in HICs: 2.8 versus 0.7 per 1000 births.^33^ In any setting, early recourse to hysterectomy when medical, non‐surgical, and conservative surgical approaches fail, is often life‐saving.

### 4.1 Strengths and limitations of the study

This study explored global and regional differences in prevalence, treatment options, and PPH‐related mortality, from the perspectives of practitioners in over 60 countries in six regions of the world, representing more than 60% of WATOG member countries. With just 5 years to the SDGs target year of 2030, achieving a global MMR of less than 70/100 000 births requires targeted interventions based on reliable data. This study represents a comprehensive effort in this direction, taking cognizance of the fact that PPH remains the leading cause of maternal mortality globally. The high response rate of over 300 within 2 weeks of the survey signifies the interest and awareness among maternity care providers to the burden of PPH and a willingness and eagerness to control it.

There is a possibility of recall and information bias, as the reported findings were based solely on the report and memory of the respondents. Findings may not truly represent the complete situation in countries and regions, as respondents came from a fraction of practitioners and hospitals in each country and region. Moreover, due to the distribution strategy used for the questionnaire, it was not possible to determine the response rate, again limiting our ability to assess whether the study sample is truly representative. These limitations notwithstanding, our study highlights concerning disparities and significant potential for improvement that should be addressed at the national, regional, and global levels.

## 5 CONCLUSIONS AND RECOMMENDATIONS

PPH remains a frequent obstetric complication with significant disparities in frequency, available treatment, and related mortality between LMICs and HICs, as well as between regions. Although oxytocin and misoprostol are widely available in all settings, their effectiveness in many LMICs may be suboptimal, owing to poor storage conditions and adulteration. Alternative uterotonics such as carboprost are not readily available due to high cost, necessitating reconsideration of the role of the cheaper heat stable carbetocin.

The NASG, an effective temporizing measure, is suboptimally distributed and thus not readily available in many LMICs where they are needed the most. Conservative surgical treatment options for intractable PPH, including skills for vascular ligation and embolization techniques, are also not commonly available in many LMICs, making radical surgery, such as hysterectomy, the primary treatment for intractable PPH in these settings.

WATOG's findings highlight the pressing need for regional and global collaboration, a multisectoral approach to improve the situation in LMICs, and to address the issues in managing PPH, to reduce maternal mortality. There is a clear need for widespread capacity building on PPH care bundles, including E‐MOTIVE,^34^ as well the management of refractory PPH, and to provide supportive healthcare systems, especially in LMICs. WATOG and FIGO's Childbirth and PPH Committee call for urgent action accordingly.

## AUTHOR CONTRIBUTIONS

AEU and AW conceptualized and designed the study and questionnaire. All authors contributed to dissemination of the study questionnaire and data collection. AEU analyzed the collected data, and together with ZM, performed the literature search and wrote the first manuscript draft. JB‐K, DS, ZJM, FB, IN, and AW critically reviewed and revised the first manuscript draft for sound intellectual content. AW coordinated the reviews and revisions, which were implemented by AEU. All authors read and approved the final manuscript.

## FUNDING INFORMATION

No funding was received for this study.

## CONFLICT OF INTEREST STATEMENT

The authors have no conflicts of interest.

## Data Availability

Data sharing is not applicable to this article as no new data were created or analyzed in this study.
